# *Breviconia acuminata* sp. nov. (Crustacea, Copepoda, Harpacticoida, Ancorabolidae), a rare benthic copepod species with a small distribution range at the tip of the Antarctic Peninsula (Southern Ocean), including some phylogenetic remarks

**DOI:** 10.3897/zookeys.1282.190452

**Published:** 2026-06-12

**Authors:** Jan Christoph Nierste, Gritta Veit-Köhler, Kai Horst George

**Affiliations:** 1 Senckenberg am Meer, German Centre for Marine Biodiversity Research DZMB, Südstrand 44, D-26382 Wilhelmshaven, Germany German Centre for Marine Biodiversity Research DZMB Wilhelmshaven Germany

**Keywords:** Endemic, marine invertebrates, Maxillopoda, meiofauna, phylogeny, Polar regions, variability

## Abstract

Meiofauna samples were collected using the multicorer during three FS POLARSTERN research expeditions to the Southern Ocean between 2013 and 2019. The samples yielded, among other representatives of the CopepodaHarpacticoida, 11 individuals of a previously unknown species of the Ancorabolidae Sars. The six males and five females could be assigned to the genus *Breviconia* Conroy-Dalton & Huys, within which they form a separate species. *Breviconia acuminata***sp. nov**. differs from the two already known species *B. australis* (George) from the Beagle Channel (Chile) and *B. andrei* Garlitska, George & Chertoprud from the Barents Sea (Russia) in the following autapomorphies: (i) the female antennule has only nine setae on the last segment instead of ten, (ii) the inner apical seta on P2enp2 is strongly shortened and only reaches half the length of the outer apical seta. In addition to the detailed description of the new species, a morphologically based comparison with the taxa *Arthropsyllus* Sars and *Uptionyx* Conroy-Dalton & Huys confirmed the monophyletic status of *Breviconia*. A key to the *Breviconia* species is also provided. *Breviconia acuminata***sp. nov**. has a very limited distribution range. To date, it has only been found on the continental shelf at the northernmost tip of the Antarctic Peninsula and on the continental slope in Bransfield Strait, at depths between 420 and 750 metres. The sediments at the stations where the new species was found were characterised by low bottom temperatures, a high silt and clay content (84–89%), and high pigment content, indicating a high food availability.

## Introduction

Benthic studies conducted in remote areas such as the deep sea or polar regions often reveal new species. Sampling is particularly challenging and costly in the Southern Ocean, so it is important that the valuable samples are used to their full potential. The sediment samples investigated for the new copepod species presented here were originally collected for ecological studies and experiments during three RV POLASTERN expeditions to the Antarctic Peninsula and the north-western and south-eastern Weddell Sea (PS 81, Hauquier and Veit-Köhler 2013; PS 96, Link et al. 2016; PS 118, Witte et al. 2019). Apart from their use in community analyses and future genetic studies, we firmly believe that the collected specimens should be utilised as extensively as possible, including for taxonomic and systematic research. Furthermore, when available, biogeographical data should be gathered for a new species to indicate where it was and was not found within an area that can be interpreted as a unit, such as the Antarctic continental shelf of the Weddell Sea and the adjacent Antarctic Peninsula in our case. Reporting on thorough sampling efforts and demonstrating where a species was not found supports the hypothesis of a limited distribution range (Mathiske et al. 2021). Ecologically, it makes sense to include material from all three expeditions in this study, as ecologically similar areas with low food availability were found in the oceanic regions of the Drake Passage (PS 81), as well as in the south-eastern Weddell Sea (PS 96), despite the clear differences in ice-cover patterns (Säring et al. 2022a). Two new infaunal species have already been described from the benthic organisms extracted from the collected sediment samples (Säring et al. 2022a; Corus et al. 2024): *Anobothrus konstantini* Säring & Bick, 2022 (Polychaeta, Ampharetidae) and *Loxosomella sigridae* Corus & Veit-Köhler, 2024 (Entoprocta, Loxosomatidae). Here we present a third new species: a harpacticoid copepod belonging to the family Ancorabolidae Sars, 1909 that we found in our material.

The taxon *Breviconia* Conroy-Dalton & Huys, 2000 was established by Conroy-Dalton and Huys (2000) to transfer the species *Arthropsyllus australis* George, 1998, described from the Beagle Channel (Chile) by George (1998), and the species *Laophontodes echinatus* Brady, 1918, described by Brady (1918) from Antarctica. While George (1998) considered the species he had described to be a sister species to *Arthropsyllus serratus* Sars, 1909, described by Sars (1909) from Norway, Conroy-Dalton and Huys (2000) had some doubts about this hypothesis. Their objections included alleged inaccuracies in George’s (1998) description – the fact that the two endites of the maxilla have only two setae instead of the three usually observed in Ancorabolinae was assumed to be an inaccurate observation made by George (1998) – and the great zoogeographic distance between the two species, which in their opinion made such a close relationship very unlikely. These objections were refuted by Garlitska et al. (2022), who described *Breviconia andrei* Garlitska, George & Chertoprud, 2022 from the Arctic Bering Sea, thereby also dispelling doubts about the accuracy of George’s (1998) description: *B. andrei* also bears only two setae on the two endites of the maxilla.

The discovery of the Arctic species enabled Garlitska et al. (2022) to establish *Breviconia* as a monophylum. Conroy-Dalton and Huys (2000) had been unable to do so because Brady (1918) had provided only a very fragmentary description of *Breviconia echinata* (Brady, 1918), and the type material – consisting only of the female holotype – no longer exists (Conroy-Dalton and Huys 2000: 373).

The 11 specimens discovered in the Antarctic show great morphological similarity to *Breviconia australis* (George, 1998), which was found ca 1,140 km northwest of the Antarctic Peninsula in the Beagle Channel (Magellan Region, Chile) (George 1998). However, they differ in some of the autapomorphies of the genus listed by Garlitska et al. (2022). In the present study, the relationships of the new species to the two known *Breviconia* species were investigated (*B. echinata* was not considered for the reasons mentioned above). In addition, an attempt was made to investigate the relationship of *Breviconia* to other representatives of the Ancorabolinae. For this purpose, we selected the monotypic genera *Arthropsyllus* Sars, 1909 and *Uptionyx* Conroy-Dalton & Huys, 2000. The taxa *Ancorabolus* Norman, 1903 and *Juxtaramia* Conroy-Dalton & Huys, 2000 show considerable deviations and were therefore not considered.

In addition to a detailed description of *Breviconia acuminata* sp. nov. and a phylogenetic classification of the new species within *Breviconia*, an identification key for this genus is presented.

## Materials and methods

### Study areas, sampling, and sample treatment

**Figure 1.**
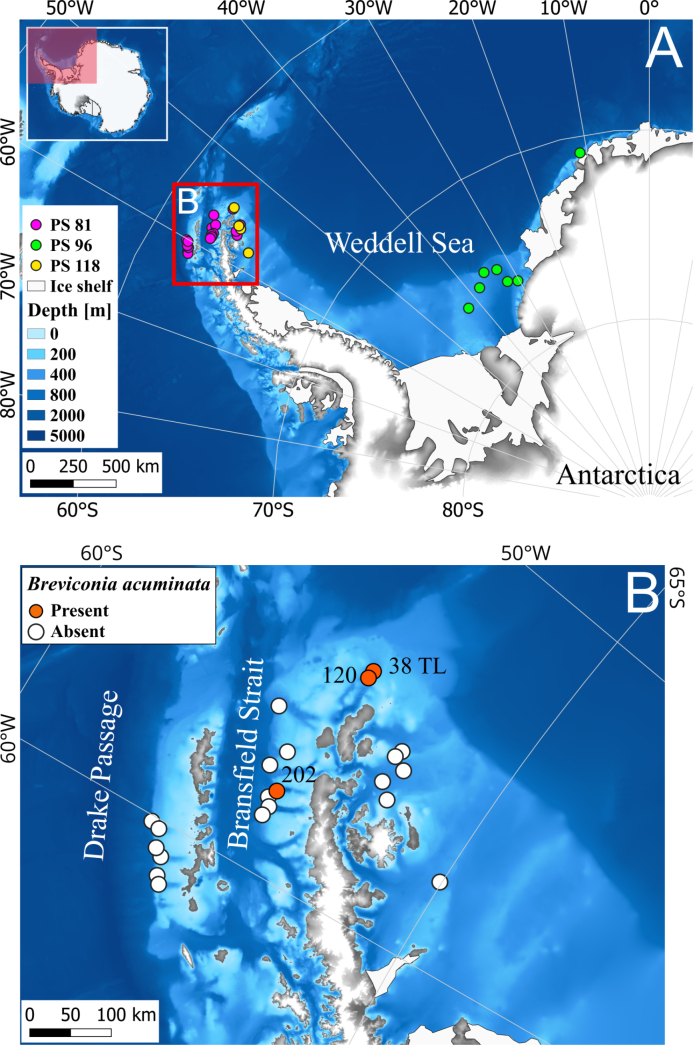
Locations of all examined stations from three RV POLARSTERN expeditions. **A**. Expeditions PS 81 (2013), PS 96 (2016), and PS 118 (2019) to the Antarctic Peninsula (with stations in Drake Passage, Bransfield Strait, and the north-western Weddell Sea) and to the south-eastern Weddell Sea; **B**. Stations in the vicinity of the Antarctic Peninsula where *Breviconia acuminata* sp. nov. was found. Abbreviation: TL type locality.

Meiofauna samples were collected from the Antarctic shelf during RV POLARSTERN expeditions PS 81 (22.01–18.03.2013; Hauquier and Veit-Köhler 2013), PS 96 (06.12.2015–14.02.2016; Link et al. 2016) and PS 118 (09.02.–10.04.2019; Witte et al. 2019). Target regions of these expeditions were the Antarctic Peninsula with stations in Drake Passage, Bransfield Strait and the north-western Weddell Sea as well as the south-eastern Weddell Sea, particularly the Filchner Trough region (Fig. 1A). Maps were created with QGIS version 3.34.14 “Prizren” with the 2024 gridded bathymetric dataset (GEBCO Compilation Group 2024) from the General Bathymetric Chart of the Ocean (GEBCO). The shelf-ice data came from the 1:50 physical vectors provided by Natural Earth (naturalearthdata.com). The coordinates of all examined stations were taken from Säring et al. (2022b) and Witte et al. (2019).

Sediment cores were taken with multiple corers (MUC; cf. Barnett et al. 1984) with differing internal diameters and surface areas of the cores (PS 81: MUC6, 57 mm, 25.5 cm^2^; PS 96 and PS 118: MUC10, 94 mm, 69.4 cm^2^). Depending on experiments conducted on board, small subsamples were taken from the cores, which reduced the sampled surface layer (PS 96, PS 118). The cores from PS 96 were sliced in 1-cm steps down to a sediment depth of 5 cm or in layers of 0–2 cm and 2–5 cm (Link et al. 2016). Cores from PS 81 and PS 118 were sliced in five 1-cm steps (Hauquier and Veit-Köhler 2013; Witte et al. 2019). All samples were preserved with a borax-buffered 4% formaldehyde-seawater solution.

In order to separate the meiofauna from the sediment, the samples were first washed with filtered tap water through a 32-µm mesh sieve and then centrifuged (Säring et al. 2022b). A colloidal silica polymer (H.C. Stark, Levasil 200/40%, ρ = 1.17) was used as a flotation medium. Kaolin was added to prevent the heavier particles from being decanted with the supernatant. After centrifugation (6 min at 4,000 rpm), the floating matter containing meiofauna organisms was decanted over the sieve and rinsed with tap water. This procedure was repeated 3 × (centrifuge method modified after Pfannkuche and Thiel 1988).

The meiofauna was sorted and counted at a higher taxon level (e.g. Annelida, Copepoda, Nematoda) using a Leica MZ 12.5 stereomicroscope. Copepods were transferred to a mixture of glycerine and water (1:1). All other taxa stayed in the sample and were preserved with 5% buffered formalin. Specimens of the new species were then spotted among the copepods with the stereomicroscope and identified with the aid of a Leica DMR microscope. Individuals belonging to *Breviconia acuminata* sp. nov. were collected from three stations on the Antarctic shelf and slope at the tip of the Antarctic Peninsula (Table 1, Fig. 1).

**Table 1.** Sampling localities where *Breviconia acuminata* sp. nov. was found. Sediment samples were collected with multicorers (MUC) during RV POLARSTERN expeditions PS 81 and PS 118 in the north-western Weddell Sea and Bransfield Strait. Station, deployment and core number, sampling date, coordinates, and water depth are given. Individual densities of *Breviconia acuminata* sp. nov., Copepoda and Nematoda are expressed per 10 cm^2^ (0–5 cm sediment depth), and sediment horizons in which the new species was found are indicated (horizon). In the following, the combined identifier “station-deployment-core” number is used. TL = Type locality; repl. = replicate (number assigned to resp. core in data base).

Expedition	Station no.	Deployment no.	Core no.	Sampling date	GPS Coordinates	Depth [m]	*Breviconia acuminata* sp. nov. [10 cm^-2^]; total (horizon)	Copepoda [10 cm^-2^]	Nematoda [10 cm^-2^]
PS 81	120	5	1	28.01.2013	63°04.58'S, 54°31.00'W	504	0.8; 2 (0-1,1-2 cm)	916.5	5482.3
PS 81	202	5	36 (repl. 3)	27.02.2013	62°55.99'S, 58°00.61'W	757	0.8; 2 (1-2 cm)	140	6194.5
PS 118	38 TL	3	16	22.03.2019	63°04.48'S, 54°20.23'W	427	0.2; 1 (1-2 cm)	940.4	4582.5
PS 118	38 TL	3	21	22.03.2019	63°04.48'S, 54°20.23'W	427	0.9; 6 (0-1 cm)	926.4	3348.9

The examined specimens are registered and deposited in the Senckenberg Forschungsinstitut und Naturmuseum Frankfurt (**SMF**), Germany. All specimens were collected with multicorers from stations on the Antarctic shelf at the tip of the Antarctic Peninsula (Table 1). Information on sampling location indicate “Locality; geographic position; water depth; expedition, sampling date, station-deployment-core number (sediment horizon); collection number”. Paratypes are numbered and described separately.

### Specimen line drawing and imaging

Holotype and paratype 1 (“allotype”) of *Breviconia acuminata* sp. nov. were selected, mounted in glycerine on slides and drawn from the dorsal view using a Leica DMR microscope equipped with differential interference contrast (DIC) and a camera lucida. Body parts (some after dissection) were drawn at magnifications up to 1600×. To ensure a high resolution, the illustrations were traced, using a Wacom Cintiq 16 drawing tablet and the software InkScape v. 1.3.2.

Selected specimens were imaged with a confocal laser-scanning microscope (CLSM). Individuals were stained with a watery solution of acid fuchsin and Congo red (modified after Michels and Büntzow 2010) and scanned with a Leica CTR 5000 (DZMB, Senckenberg am Meer) equipped with a Leica DM 5000 B microscope (400× magnification) and a visible light laser (DPSS561). Using the image stacks, maximum intensity projections were created with the Leica LAS software (Leica Microsystems). The images were assembled and adjusted for colour and brightness using the software Affinity v. 3.0.2.3912 (Canva Pty Ltd.).

### Abbreviations used in the text

**A1** antennule;

**A2** antenna;

**aes** aesthetasc;

**cphth** cephalothorax;

**enp1–3** first–third segment of the endopod;

**exp1–3** first–third segment of the exopod;

**FR** furcal ramus/rami;

**GDS** genital double somite;

**md** mandible;

**mxl** maxillula;

**mx** maxilla;

**mxp** maxilliped;

**P1–P6** swimming legs (pereiopods) 1–6.

### Environmental characteristics

Water-column parameters were obtained from Schröder et al. (2013) and Janout et al. (2020). Sediment parameters were determined according to Säring et al. (2022b) and obtained from Vanreusel et al. (2021) and Weith et al. (2023). For the present study, the following environmental parameters were relevant: *Water column* – temperature near the sea bottom (*_bottom_*T), salinity near the sea bottom (*_bottom_*Sal); *sediment* – chlorophyll *a* content (_*sed*_Chl *a*), phaeopigment content (_*sed*_Phaeo), carbon:nitrogen ratio, content of silt and clay (<63 µm), sand (63–500 µm), and coarse sand (>500 µm). Values were averaged across the 0–5 cm layers of sediment cores in which *Breviconia acuminata* sp. nov. specimens were found. Otherwise, values from cores from the same deployment are presented.

## Results

### Taxonomy

**Order Harpacticoida Sars, 1903**

**Family Ancorabolidae Sars, 1909**

**Subfamily Ancorabolinae Sars, 1909**

#### Genus Breviconia

Taxon classificationAnimaliaHarpacticoidaAncorabolidae

Conroy-Dalton & Huys, 2000

A1ED9AE4-5DD5-583D-ADF1-E3BBA6FA40FB

##### Species.

Species *Breviconia acuminata* sp. nov., *B. australis* (George, 1998) (type species) (syn. *Arthropsyllus australis* George, 1998), *B. andrei* Garlitska, George & Chertoprud, 2022; *species inquirenda*: *B. echinata* (Brady, 1918) (syn. *Laophontodes echinatus* Brady, 1918).

##### Generic diagnosis.

Ancorabolidae Sars, 1909, Ancorabolinae Sars, 1909. Body slightly depressed dorsoventrally, slightly tapering posteriorly, without clear demarcation between pro- and urosoma; sexual dimorphism in the antennule, P3 and P4 endopod, P5 and P6; females with fused last thoracic and first abdominal somites forming a genital double somite. Cphth with moderately developed sensillar groups I–V; sensillar group V with backwardly directed tooth-like or conical cuticular processes. Rostrum narrowed, fused to cphth, with pair of sensilla and tube pore. Body somites except penultimate one and telson with lateral and dorsolateral cuticular processes; P2–P5-bearing body somites additionally with dorsal processes of varying size. Processes ending in cup-shaped tips that carry one sensillum, which inserts like a ball-and-socket joint and presents a special shape, i.e. a broad and tapering spiniform proximal half, combined with a flagelliform distal half. Female antennule 3-, male antennule 7-segmented, subchirocer, with swollen fourth segment; segments of female antennule densely covered with minute spinules or smooth; A2 with allobasis that bears two abexopodal setae; exopod missing; endopod anteriorly with two spines and one slender seta; apically with one short spine and five setae, three of which are geniculate, and one very short. Mandibular palp carrying all six setae or lacking one seta (corresponding to the originally proximal basal seta). Coxa of the maxillula with one or two apical setae; basis, endo- and exopod fused to single lobe that bears eight setae/spines. Maxilla with two endites, the proximal one with two or three, the distal one with two apical elements; allobasis produced into strong claw that is basally accompanied by three setae; endopod completely reduced and only represented by two setae. Maxilliped prehensile, with syncoxa lacking any seta; allobasis slightly longer than syncoxa; endopod produced into long claw that is or not accompanied by minute seta basally. Swimming legs with small coxae, bow-like intercoxal sclerites, transversely elongated bases and 2-segmented endopods. P1enp1 at least as long as or clearly longer than whole exopod, unarmed; enp2 much shorter than enp1, with one subapical inner seta and two apical long geniculate setae: Exopod 2-segmented, exp1 with one outer spine, exp2 with two outer spines and three geniculate setae. P2–P4 with 3-segmented exopods showing the setal formula I, I-1, II-2-0; enp1 small, unarmed, enp2 much longer than enp1; female P2–P4enp2 with one inner and two apical setae; female P3 and P4 additionally with one outer seta; male P2 as well as P3 and P4 exopods as in female; male P3 endopod 2- or 3-segmented, first segment small, second segment longest, with strong curved apophysis on the inner edge; when the endopod is 3-segmented, enp3 is small and bears two apical setae; in case of a 2-segmented endopod, the former enp3 is fused to enp2 that then carries the two apical setae distally from apophysis. P5 with baseoendopod and distinct exopod; baseoendopod with a long setophore that bears one seta. In the female the endopodal lobe is elongated and equipped with two inner and two apical setae; instead in the male it is much shorter or even almost lost and carries two apical elements. Likewise, the female P5 exopod is larger than the male’s, but in both sexes the exopod bears two outer setae/spines, two apical setae, and one inner serrate seta. Female P6 vestigial, forming genital operculum, each lobe with two or three small setae. Furcal rami slightly trapezoid or cylindrical and slender in dorsal view, ~ 3–7× longer than broad, with seven setae (a re-examination of the furca of the holotype of *B. australis* revealed that all seven furcal setae are present. Seta I is tiny and located directly ventral to seta II. It had been overlooked by George (1998)).

#### Breviconia acuminata sp. nov.

Taxon classificationAnimaliaHarpacticoidaAncorabolidae

0625A1D2-293D-5D1E-9841-C586BC0EC272

https://zoobank.org/93F25AF9-F106-417A-AAEB-D02A72D6C7A9

Figs 1, 2, 3, 4, 5, 6, 7, 8, 9, 10, 11, 12, 13, 14, 15

##### Type material.

***Holotype***. • Adult male on one slide; collected in the north-western Weddell Sea (Antarctic shelf); 63°04.480'S, 54°20.226'W; water depth 427 m; during RV POLARSTERN cruise PS 118 on 22.03.2019 at station 38-3-21 (upper 0–1 cm); collection number SMF 37373/1. ***Paratypes***. • #1 (“allotype”): adult female on two slides; same data as for holotype; coll. no. SMF 37374/1–2. • #2: adult male on one slide; same data as for holotype; coll. no. SMF 37375/1. • #3: adult male on 10 slides; same data as for holotype; coll. no. SMF 37376/1–10. • #4: adult male on one slide; same data as for holotype but from core 16 and from sediment horizon 1–2 cm; coll. no. SMF 37377/1. • #5: adult female on one slide; same data as for holotype; coll. no. SMF 37378/1. • #6: adult male on one slide; same data as for holotype; coll. no. SMF 37379/1. • #7: adult female on one slide; north-western Weddell Sea (Antarctic shelf); 63°4.58'S, 54°31.00'W; 504 m; RV POLARSTERN cruise PS 81, 28.01.2013, station 120-5-1 (1–2 cm); coll. no. SMF 37380/1. • #8: adult female on one slide; same data as for preceding but from sediment horizon 0–1 cm; coll. no. SMF 37381/1. • #9: adult male on one slide; Bransfield Strait (Antarctic shelf); 62°55.99'S, 58°0.61'W; 757 m; RV POLARSTERN cruise PS 81; 27.02.2013; station 202-5-36 (36 = replicate 3) (1–2 cm); coll. no. SMF 37382/1. • #10: adult female on one slide; same data as for preceding; coll. no. SMF 37383/1.

##### Type locality.

North-western Weddell Sea (Antarctic shelf), geographic position 63°04.480'S, 054°20.226'W, sediment sample collected during RV POLARSTERN cruise PS 118, on 22.03.2019, station no. 38, water depth 427 m (Table 1, Fig. 1).

##### Description of adult male.

**Figure 2.**
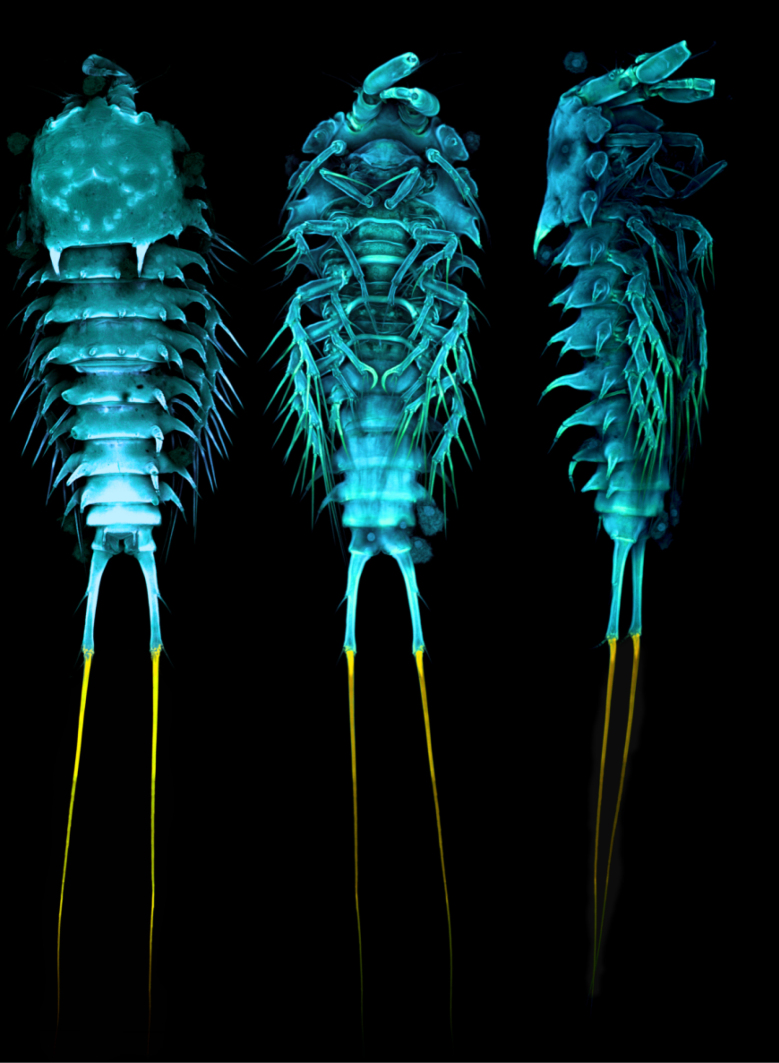
*Breviconia acuminata* sp. nov. ♂ paratype 6. CLSM image. Dorsal, ventral, right lateral view (left to right). Body length of depicted specimen 479 μm (from anterior tip of rostrum to posterior end of the furcal rami).

**Figure 3.**
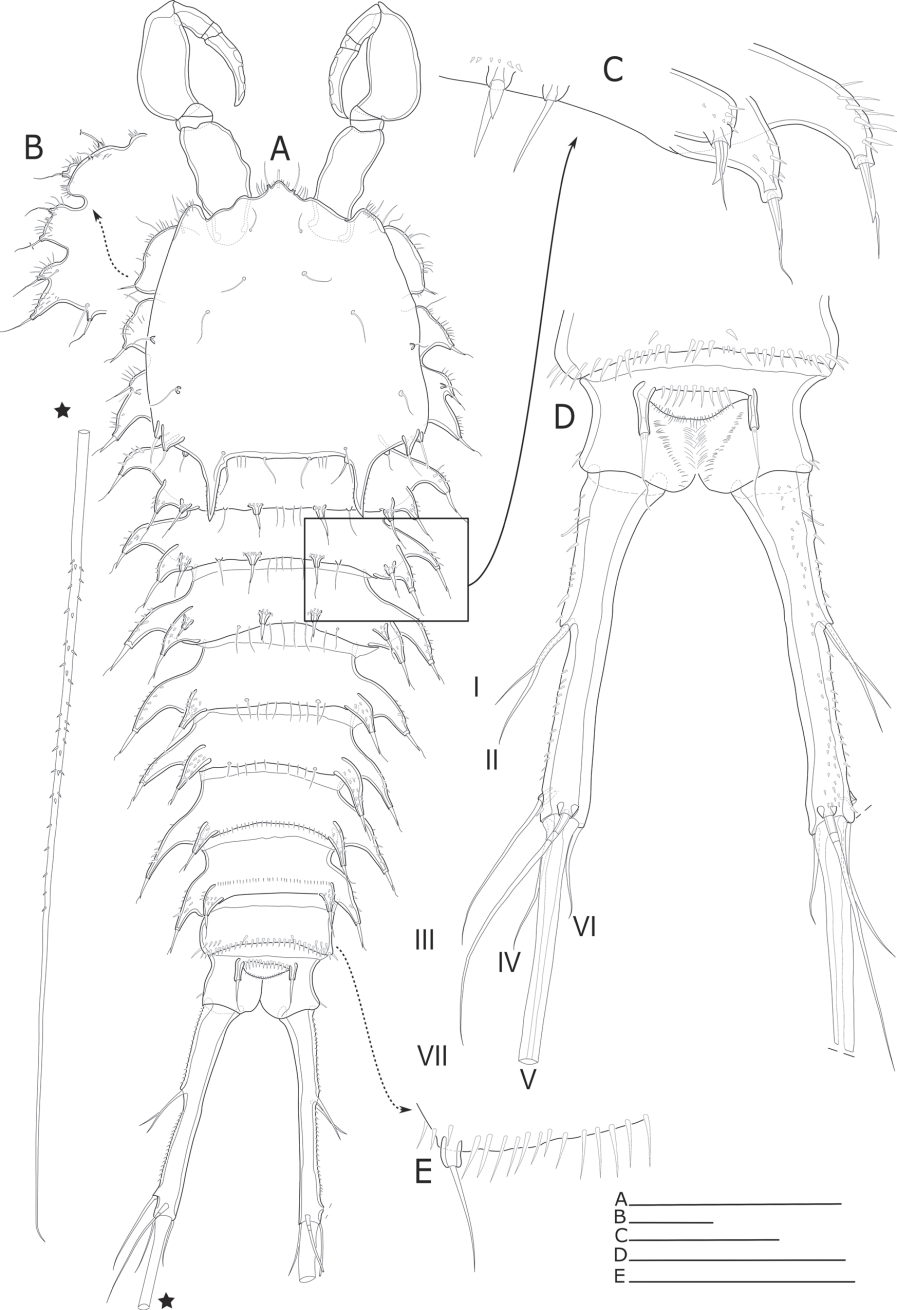
*Breviconia acuminata* sp. nov. ♂ **A**. Holotype, habitus, dorsal view (terminal seta V drawn in two parts, see star); **B**. Paratype 3, left cuticular processes of the cephalothorax; **C**. Holotype, right lateral, dorsolateral, and dorsal cuticular processes; **D**. Holotype, furcal rami, dorsal view; **E**. Paratype 2, right ventral posterior row of spinules and sensillum. Scale bars: 100 μm (**A, C**), 50 μm (**B, D**), 25 μm (**E**).

**Figure 4.**
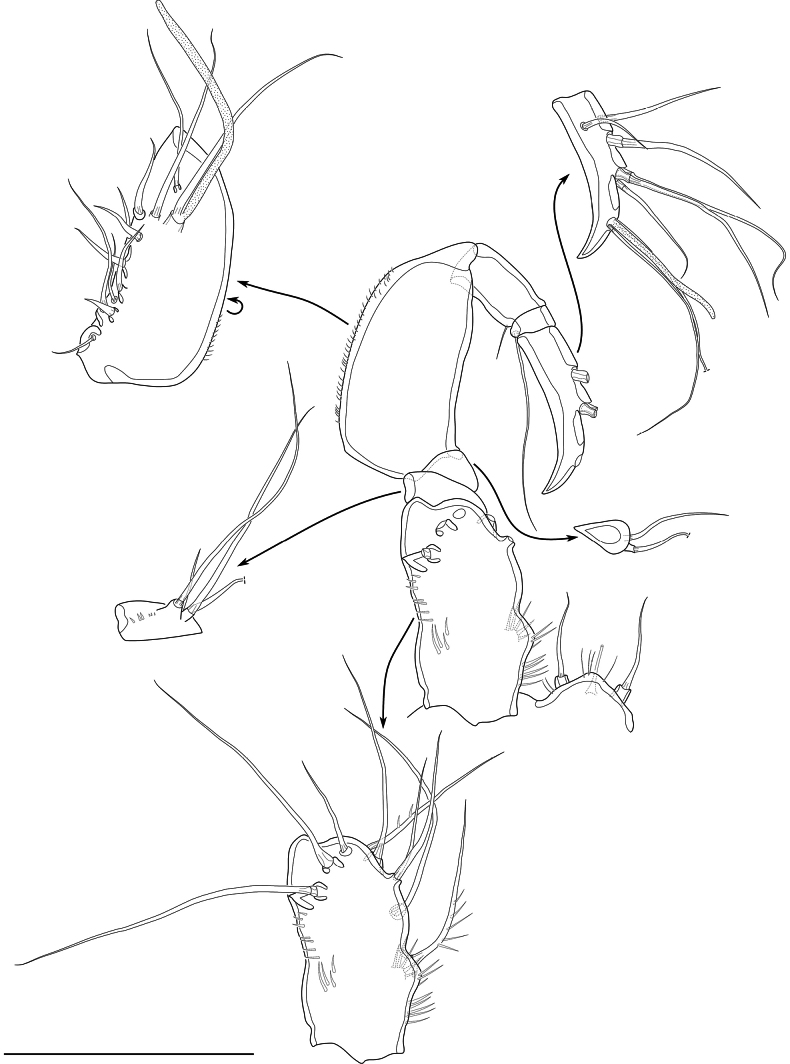
*Breviconia acuminata* sp. nov. ♂ holotype. Rostrum and left antennule, dorsal view, fourth segment, ventral view. Scale bar: 50 μm.

***Habitus*** (Figs 2, 3A). Mean total body length from anterior tip of rostrum to posterior tip of the FR: 499 μm (*n* = 5; length of holotype depicted in Fig. 2: 493 μm). Body dorsoventrally depressed, tapering posteriorly. No clear demarcation between prosome and urosome. Rostrum (Fig. 4) small, with terminal tube pore and two lateral sensilla. Cephalothorax (Fig. 3A) as wide as long with a slight increase in width from anterior to posterior. Dorsally and dorsolaterally with six pairs of sensilla. Laterally with three pairs of moderate cuticular processes bearing two sensilla (Fig. 2A, B). Posteriorly with a pair of pointed cuticular processes covered with lateral rows of spinules. Extending slightly over the first free body somite. Four sensilla between these cuticular processes. Pair of short sensilla-bearing cuticular processes at the posterior corners. Knob-like sensilla-bearing tubercles between the two aforementioned larger structures. All free somites, with the exception of the penultimate somite and telson, bearing a pair of backwards produced lateral and dorsolateral horn-shaped cuticular processes with two rows of spinules (Fig. 3A, C). Free somites bearing P2–P5 with additional pairs of dorsolateral and dorsal sensilla-bearing tubercles with one or two rows of spinules (Fig. 3A, C). Dorsal pair of sensilla on free somites bearing P2–P6. Penultimate somite with posterior row of spinules. Telson somite with lateral spinules near the base of the FR. Anal operculum dorsally with two rows of spinules between a pair of sensilla-bearing tubercles. Free somites 6–9 ventrally with row of spinules on the posterior end. Somites 6–8 additionally with a pair of tubercles bearing a terminal sensillum.

***FR*** (Fig. 3A, D) ~ 8× as long as wide. Outer lateral and dorsal surface covered with spinules. Tube pore located laterally near the base of each ramus. Inner lateral surface smooth. Position of setae: I and II laterally at half length of the ramus. I slightly shorter than II. III and VII inserting subterminally and arising from small pedestals; III longer than IV, located at the outer margin; VII second longest seta, tri-articulate and located dorsally. IV–VI arising terminally; IV approximately as long as I, at outer edge. V longest and multipinnate (drawn in two parts in Fig. 3A, see star). VI shortest, at inner edge.

***A1*** (Fig. 4) 7-segmented. First segment: inner margin with row of spinules between the base and the first seta. One group of long spinules ventrally near the base of the first seta. Six setae on the inner margin. First and last inner lateral setae pinnate. Remaining seven setae bare. Outer margin unarmed. Dorsally with one group of longer and one row of shorter spinules between the base and half-length of the segment. Pointed cuticular protrusion at the anterior end of the row of short spinules. Three bare setae of which two grow from small tubercles. Second segment: Small, vaguely rectangular in shape. Dorsal row of small spinules. Inner margin with two long setae and two short setae. Third segment: Tear-shaped. Very small, ~ 1/6^th^ of the length of the first segment. Two short setae on the inner margin, one of which growing from small tubercle. Fourth segment: Approximately as long as the first segment. Outer margin with small spinules. Ventrally with 11 bare setae, nine of which are located near the inner margin and one aesthetasc. Aesthetasc and one seta form an acrothek. Fifth segment with one ventral short bare seta near the base of the sixth segment, ~ 4× longer than the sixth segment. Sixth segment with one ventral bare seta near the base of the seventh segment. Seventh segment claw-shaped, curving slightly inwards. One dorsal and one ventral bare seta near the base of the segment, outer margin with four bare setae and one trithek containing two bare setae and one aesthetasc. Setal formula: 1-[9], 2-[4], 3-[2], 4-[10+(1+aes)], 5-[1], 6-[1], 7-[6+(2+aes)].

**Figure 5.**
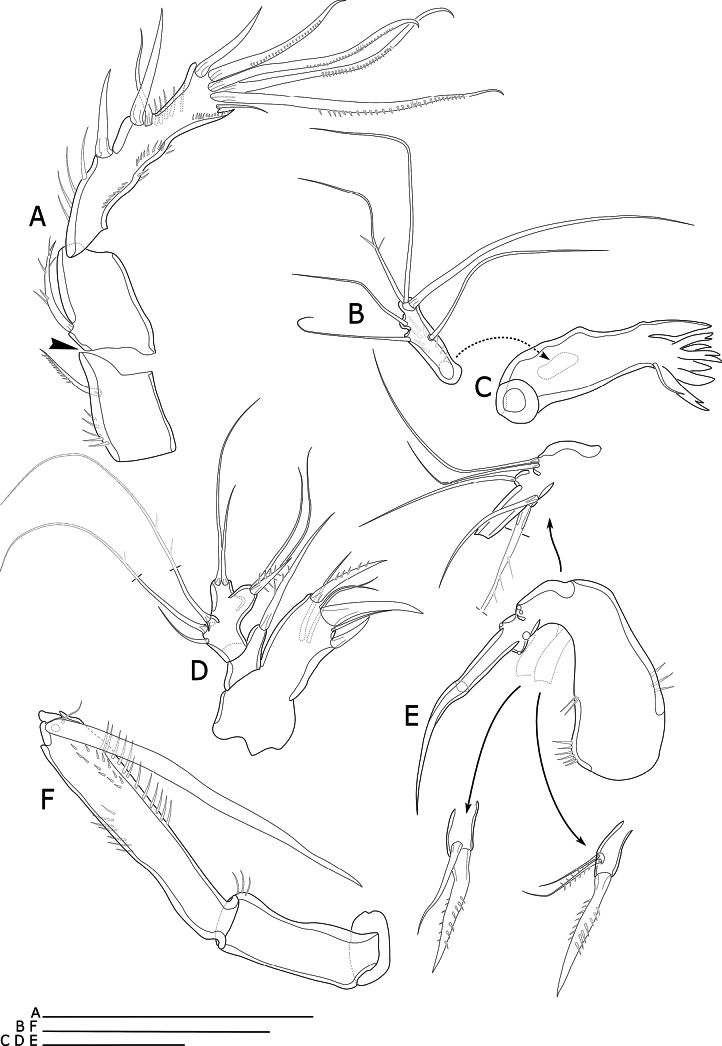
*Breviconia acuminata* sp. nov. **A**. ♂ paratype 3, right antenna with broken allobasis (arrowed); **B**. ♀ paratype 1 (“allotype”), left mandibular palpus, rows of spinules added after paratype 2; **C**. ♂ paratype 2, left mandible; **D**. ♂ paratype 2, right maxillula, posterior view; **E**. ♂ paratype 3, left maxilla, posterior view, pinnate seta on basis added after paratype 1; **F**. ♀ paratype 1, right maxilliped, posterior view. Scale bars: 50 μm (**A, B, F**); 20 μm (**C–E**).

***A2*** (Fig. 5A). Basis and first endopodal segment fused forming allobasis (broken in Fig. 5A, marked by arrow). Exopod absent. Endopod 1-segmented, approximately as long as allobasis. Outer margin with row of spinules near the base, one pinnate and one bipinnate abexopodal seta. Inner margin smooth. Endopod with rows of spinules on both outer margins. Outer margin with three bare setae. Terminally with four pinnate setae, three of which geniculate, and two bare setae, one of which very short.

***Md*** (Fig. 5B, C). Coxa unarmed. Gnathobase with seven teeth and one small, bare seta. Basis, endopod and exopod fused into 1-segmented mandibular palp carrying two rows of spinules on the surface and six setae.

***Mxl*** (Fig. 5D). Arthrite of praecoxa terminally with two slender and three strong setae, one of the latter ones pinnate. Subapically with two slender bare setae. Coxa with one pinnate seta. Basis, endopod and exopod fused into 1-segmented palp with two endites. Proximal endite with two long, bare and one very short bipinnate seta. Distal endite with one long and one short bare seta. Endopod represented by one long pinnate seta. Exopod represented by one short bare and one long pinnate setae.

***Mx*** (Fig. 5E). Syncoxa with spinules on both margins and two endites. Proximal endite with one terminal bipinnate spine, one bare and one bipinnate setae. Distal endite with terminal bipinnate spine and one bare seta. Basis fused with syncoxa bearing one claw-like seta fused to basis, two bare setae, and one pinnate seta. Endopod fused to basis, represented by two bare setae.

***Mxp*** (Fig. 5F) prehensile, syncoxa terminally with spinules. Basis with rows of spinules on all surfaces. Endopod transformed into large claw, ~ 1.25× as long as the base, with one accompanying minute seta.

**Figure 6.**
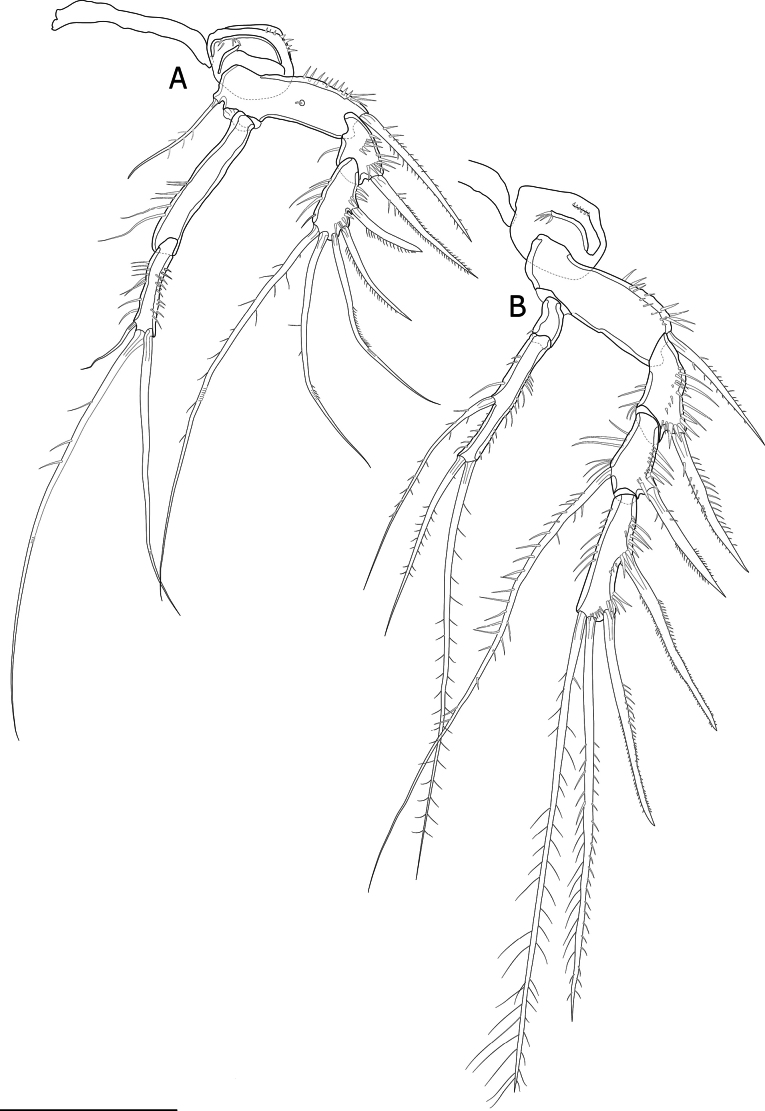
*Breviconia acuminata* sp. nov. ♂ paratype 2; **A**. Left P1, anterior view; **B**. Left P2, anterior view. Scale bar: 50 μm.

***P1*** (Fig. 6A). Basis transversally elongated with one bipinnate inner seta and one bipinnate outer spine. Spinules at the base of the outer spine more robust than the other spinules. Tube pore at approximately half-length of the base. Exopod 2-segmented. Exp1 with two rows of long spinules near the outer margin, one setule on the inner margin and one outer bipinnate spine. Spinules at the base of the outer spine more robust than the other spinules. Exp2 with rows of spinules on both margins, two outer pinnate spines. Terminally with three geniculate setae: one pinnate, one bipinnate and one very long bipinnate seta. Endopod 2-segmented, Enp1 twice as long as Enp2. Enp1 with row of spinules on the upper half of the inner margin. Enp2 with row of spinules on outer margin and patches of spinules on outer margin and surface. Terminally with two bare setae, one of which is very short, and one very long pinnate seta. The two long setae are geniculate.

***P2*** (Fig. 6B). Basis transversally elongated with patches of spinules along the outer margin and one bipinnate outer spine. Exopod 3-segmented. Exp1 with two rows of spinules at the outer margin, one row of spinules at the inner margin and one bipinnate outer spine. Exp2 with two rows of spinules at the outer margin, one row of spinules at the inner margin, one outer bipinnate spine and one very long inner bipinnate seta. Exp3 with one row of spinules at the outer margin, one row of spinules at the inner margin and two outer bipinnate spines. Terminally with patches of spinules. Two terminal setae, one bipinnate and one plumose and pinnate. Endopod 2-segmented with Enp2 4× longer than Enp1. Enp1 with spinule at the inner margin, near the base of Enp2. Enp2 with rows of spinules at both margins and one outer bipinnate seta. Terminally with two bipinnate setae of unequal length.

**Figure 7.**
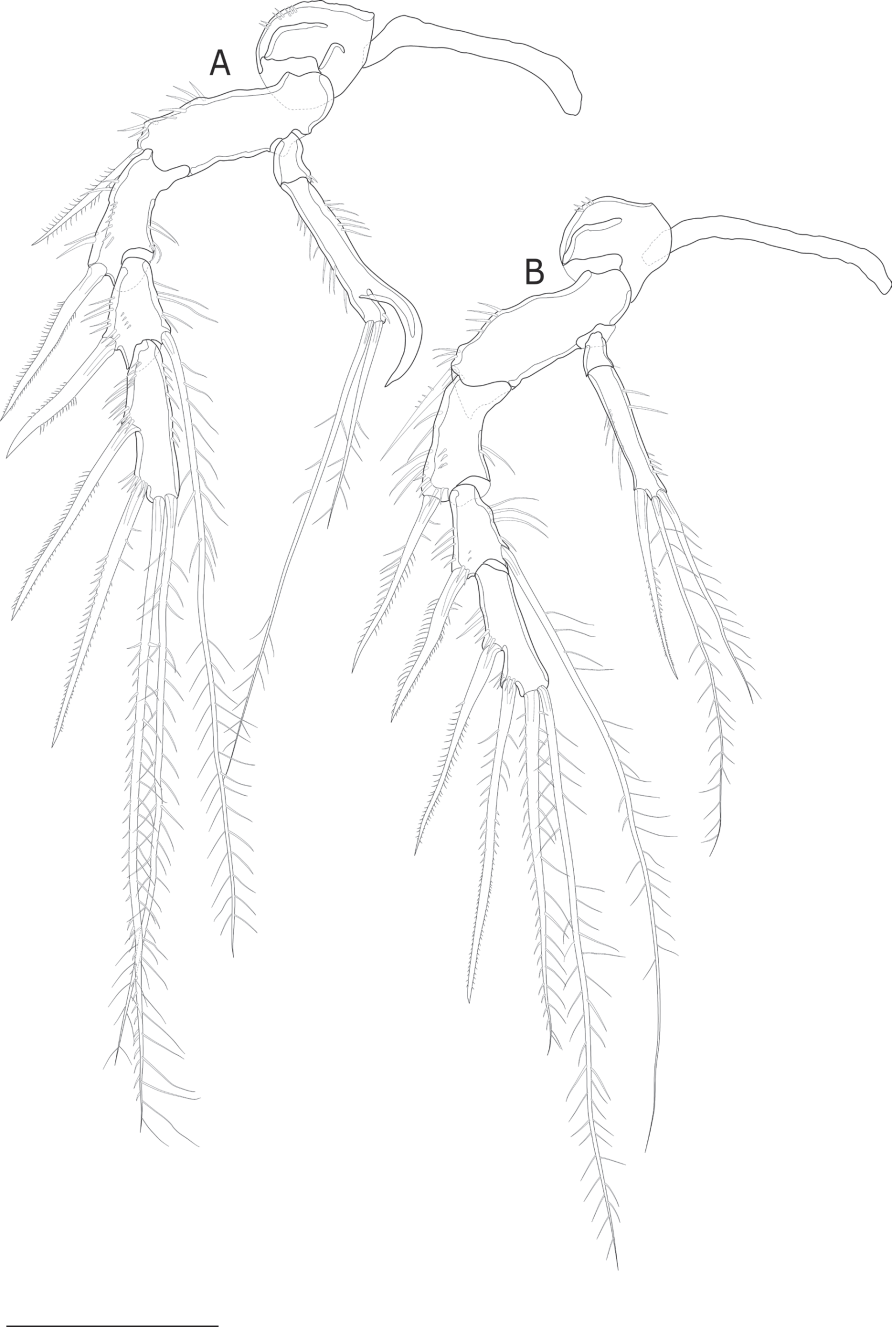
*Breviconia acuminata* sp. nov. ♂ paratype 2 **A**. Right P3, anterior view; **B**. Right P4, anterior view. Scale bar: 50 μm.

***P3*** (Fig. 7A). Basis transversally elongated with patches of spinules along the outer margin and one bipinnate outer spine. Exopod 3-segmented. Exp1 with patches of spinules at both margins and one bipinnate outer spine. Exp2 with patches of spinules at both margins, one bipinnate outer spine and one very long plumose inner seta. Exp3 with rows of spinules at both margins and two outer bipinnate. Terminally with two setae, one bipinnate and one plumose and pinnate. Endopod 2-segmented with Enp2 4× longer than Enp1. Enp1 with spinules at the inner margin, near the base of Enp2. Enp2 with row of spinules on both margins. Smooth recurved apophysis growing distally from inner margin. Terminally with two bipinnate setae of unequal length.

***P4*** (Fig. 7B). Basis transversally elongated with patches of spinules along the outer margin and one bipinnate outer spine. Exopod 3-segmented. Exp1 with patches of spinules at both margins and one bipinnate outer spine. Exp2 with patches of spinules at both margins, one bipinnate outer spine and one very long plumose inner seta. Exp3 with rows of spinules at both margins and two outer bipinnate spines. Terminally with two setae, one bipinnate and one plumose and pinnate. Endopod 2-segmented with Enp2 4× longer than Enp1. Enp1 smooth. Enp2 with row of spinules on both margins and one subterminal bipinnate outer spine. Terminally with two bipinnate setae of unequal length.

The setal formula of P1–P4 of both sexes is given in Table 2.

**Table 2.** Setal formula of swimming legs P1–P4 of *Breviconia acuminata* sp. nov. following Sewell (1949). Roman numerals: outer elements; Arabian numerals: apical/inner elements. Note the sexual dimorphisms in male and female P3 and P4.

Swimming leg	Exopod	Endopod
P1	I-0: II,3,0	0-0:0,2,1
P2	I-0: I-1: II,2,0	0-0:0,2,1
P3 male	I-0: I-1: II,2,0	0-0: 0(apophysis),2,0
P3 female	I-0: I-1: II,2,0	0-0: I,2,1
P4 male	I-0: I-1: II,2,0	0-0: I,2,0
P4 female	I-0: I-1: II,2,0	0-0: I,2,1

**Figure 8.**
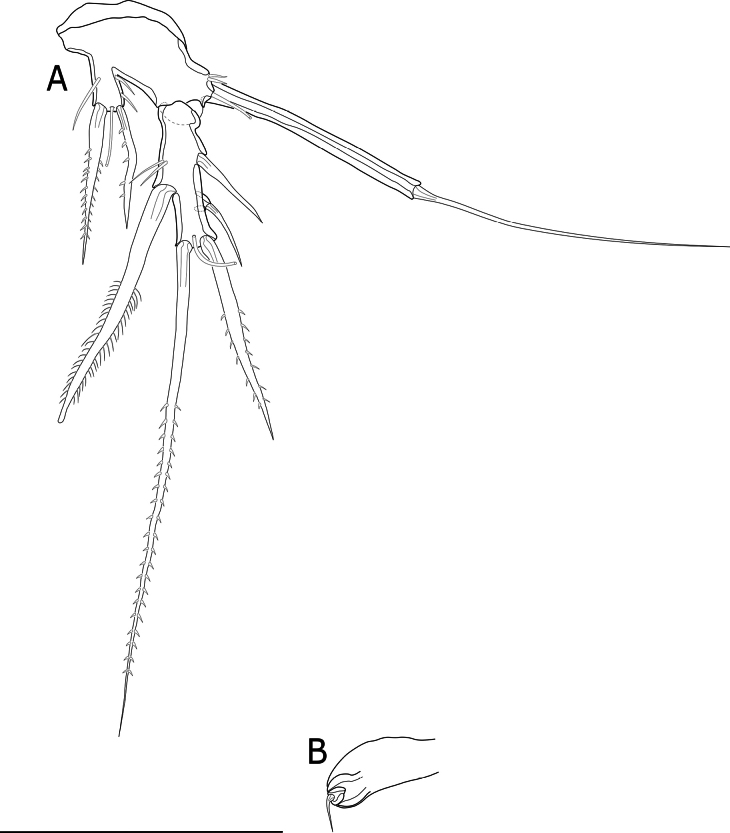
*Breviconia acuminata* sp. nov. ♂ paratype 2. **A**. Left P5, anterior view; **B**. Right P6. Scale bar: 50 μm.

***P5*** (Fig. 8A). Baseoendopod elongated with one bare outer seta arising from a long setophore which reaches 1.5× the length of the exopod. Patch of spinules and one tube pore at the base of the setophore. Endopodal lobe with isolated spinules at both margins. Terminally with one inner bipinnate spine, one outer pinnate spine and one tube pore in between the spines. Exopod 4× as long as wide, with a patch of spinules at the inner margin, one large bipinnate inner spine and two outer spines. Terminally with one long bipinnate seta, one short bipinnate seta and a tube pore in between the two setae.

***P6*** (Fig. 8B) asymmetrical, with only one functional member. Bulbous base with a small tubercle bearing a terminal bare seta. Base tapers towards the other side where it fuses with the somite.

##### Description of adult female.

**Figure 9.**
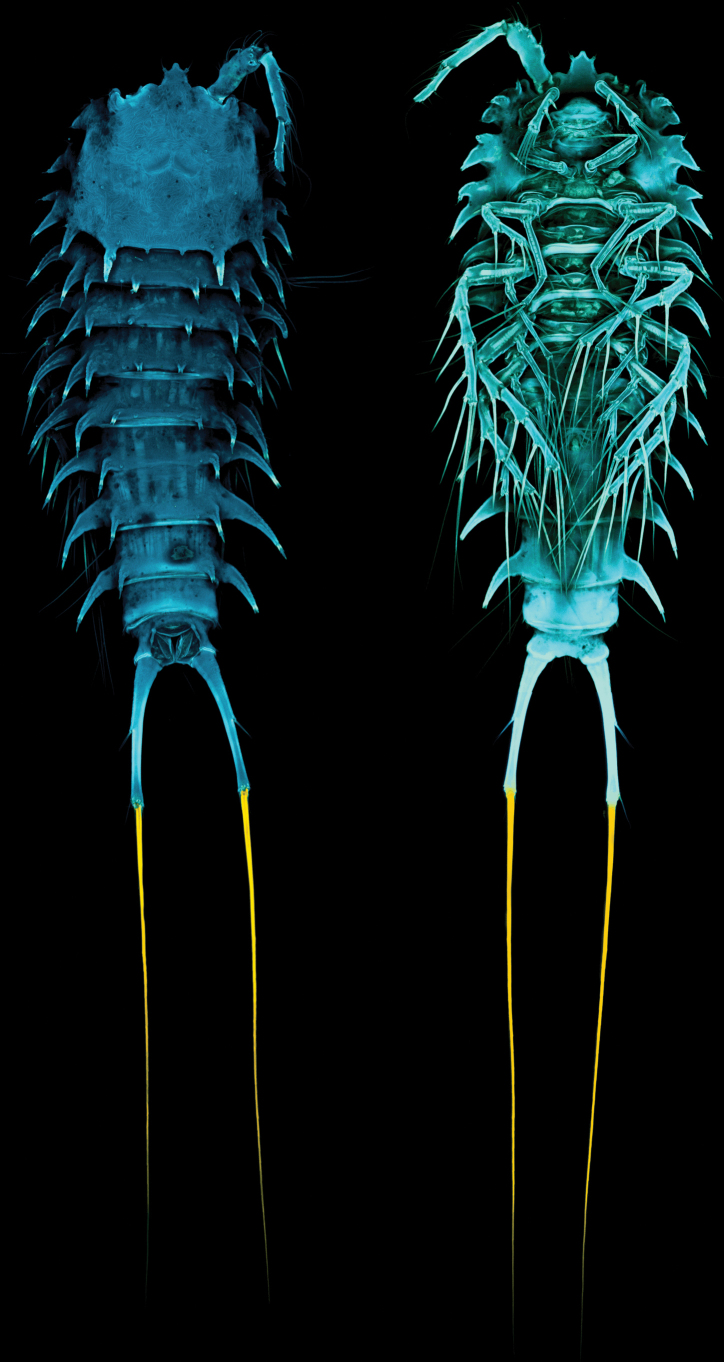
*Breviconia acuminata* sp. nov. ♀ paratype 7. CLSM image. Dorsal, ventral view (left, right). Body length of depicted specimen 841 μm (from anterior tip of rostrum to posterior end of the furcal rami).

**Figure 10.**
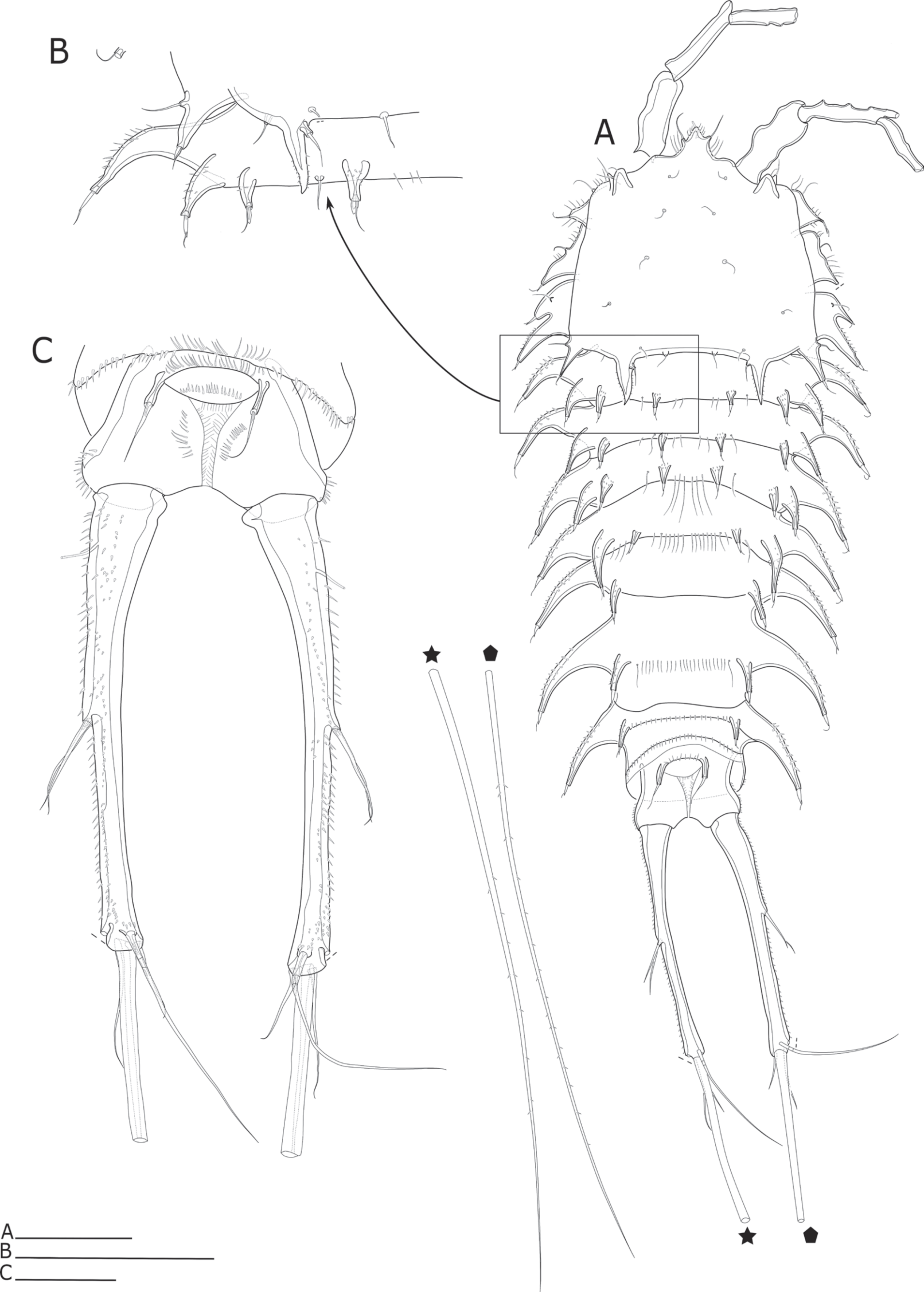
*Breviconia acuminata* sp. nov. ♀ paratype 1 (“allotype”). **A**. Habitus, dorsal view (terminal setae V drawn in two parts, see symbols); **B**. Left lateral, dorsolateral, and dorsal cuticular processes; **C**. Furcal rami, dorsal view. Scale bars: 100 μm (**A**); 50 μm (**B, C**).

**Figure 11.**
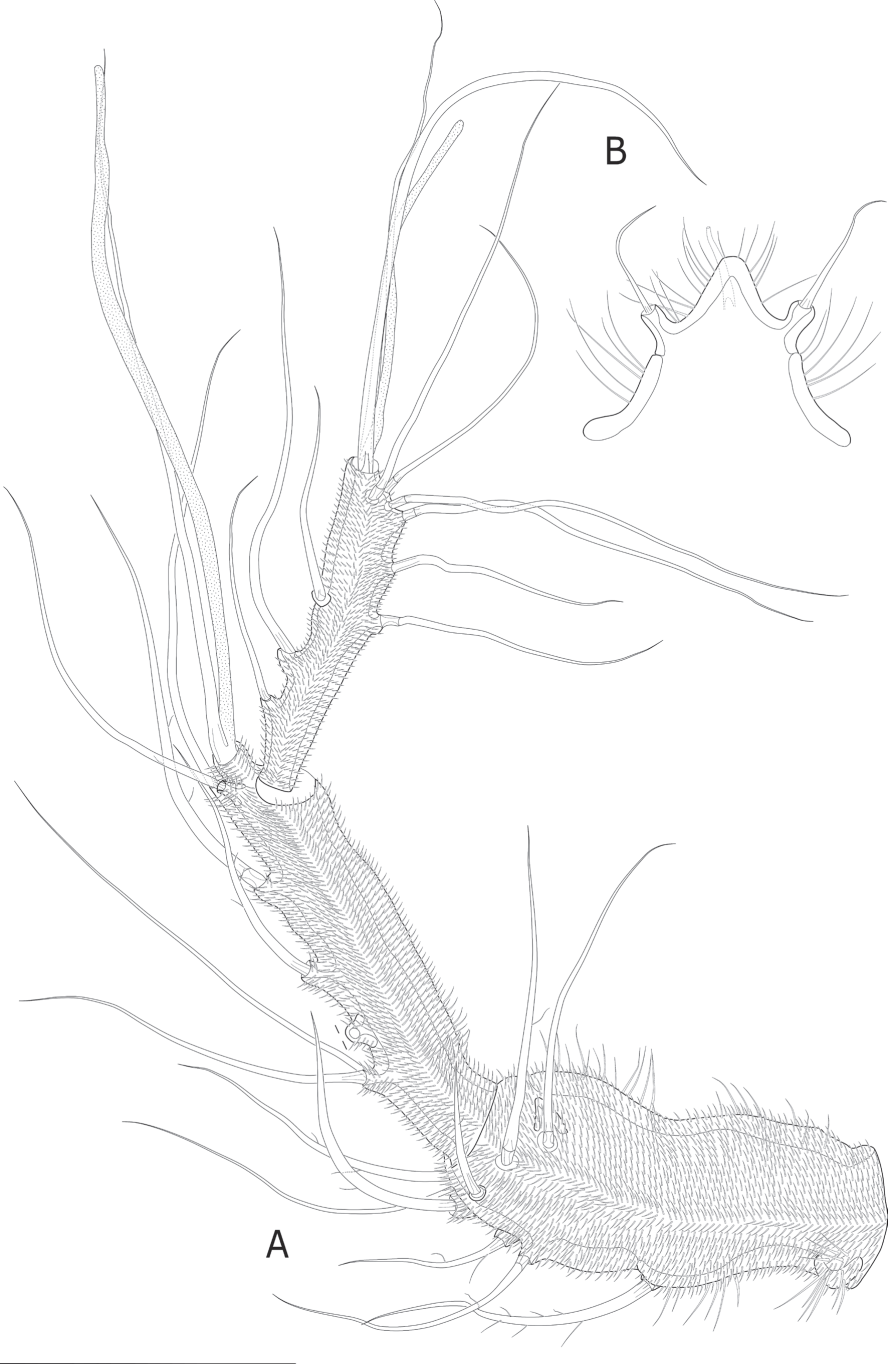
*Breviconia acuminata* sp. nov. ♀ paratype 1 (“allotype”). **A**. Right antennule, dorsal view; **B**. Rostrum. Scale bar: 50 μm.

***Habitus*** (Figs 9, 10A). Mean total body length of all examined specimens measured from anterior tip of rostrum to posterior tip of the FR: 840 μm (*n* = 5; length of paratype 1 (“allotype”) depicted in Fig. 10: 800 μm). Rostrum (Figs 10A, 11B) as in male. Single cuticular processes with one lateral and one terminal sensillum at the posterior corners of the cephalothorax. Approximately as long as the cuticular processes at the posterior margin. Last thoracic and first abdominal somite fused to form GDS. Original separation indicated dorsally by a row of spinules and the position of the lateral and dorsolateral cuticular processes. Otherwise as in male.

***FR*** (Fig. 10A, C) ~ 12× as long as wide. Armament as in male (terminal seta V drawn in two parts in Fig. 10A, see symbols).

***A1*** (Fig. 11A) 3-segmented, with dense coverage of tiny spinules on all segments. First segment: Inner margin with row of spinules. Outer margin with rows of spinules between the base and the half-point. One group of long spinules on a dorsal cuticular process at the base of the segment. No seta on the inner margin. Outer margin with six setae: one at the half-point and five distally. Dorsal surface distally with a cuticular process and three setae. Second segment: as long as first segment. No seta on inner margin. Outer margin with seven setae, three of which grow from tubercles. Distally with one long aesthetasc and one seta forming an acrothek. Third segment: slightly shorter than second segment. Inner margin with five setae. Outer margin with three setae. Distally with one dorsal seta and a trithek containing one aesthetasc and two bare setae. Setal formula: 1-[9], 2-[7+(1+aes)], 3-[9+(2+aes)].

A2, Md, Mxl, Mx, Mxp, P1, and P2 as in male.

**Figure 12.**
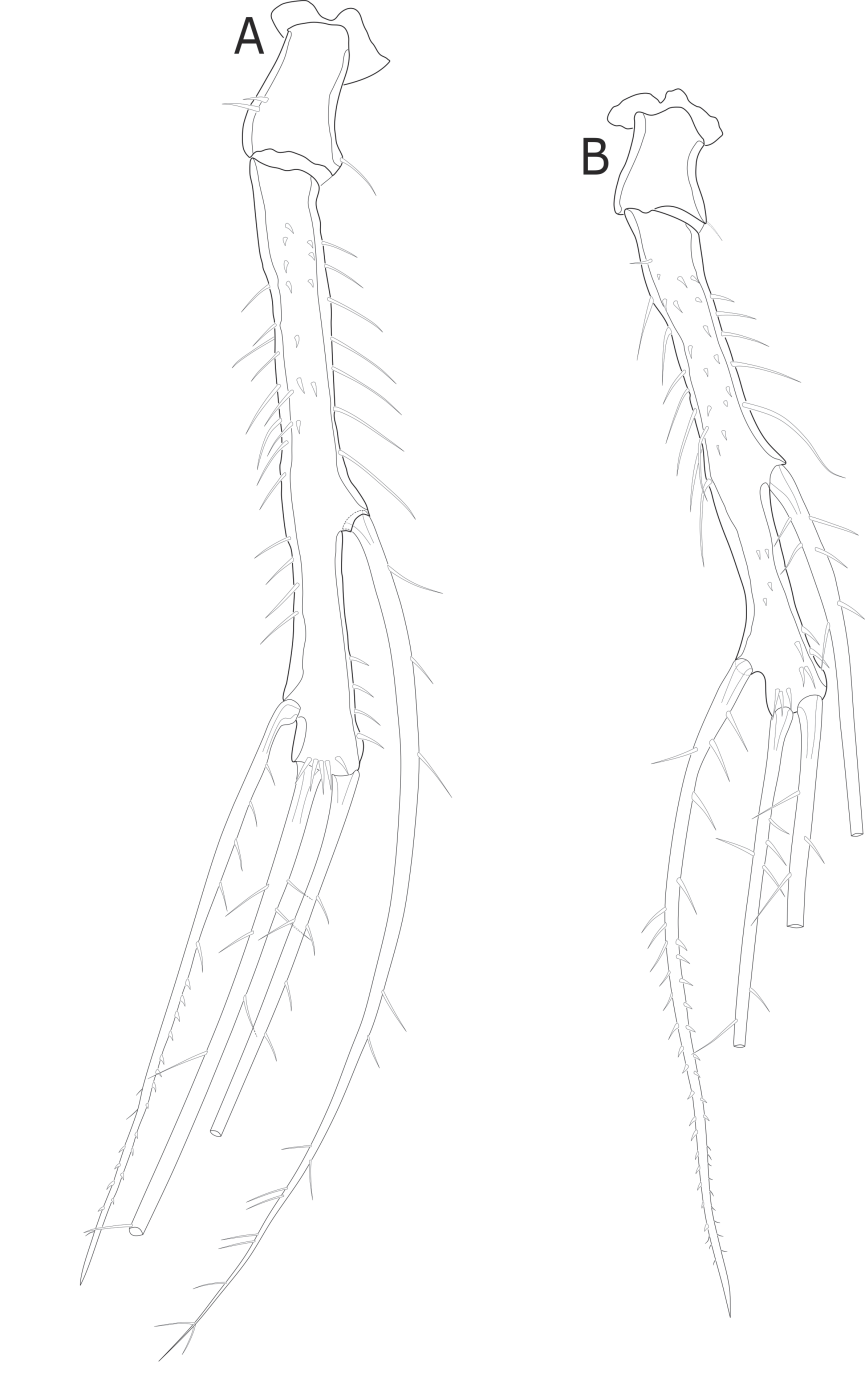
*Breviconia acuminata* sp. nov. ♀ paratype 1 (“allotype”). **A**. Right P3 endopod, anterior view; **B**. Left P4 endopod (spinule on enp1 added after right P4), anterior view. Scale bar: 50 μm.

***P3*** (Fig. 12A). Exopod as in male. Endopod 2-segmented. Enp2 4× longer than Enp1. Enp1 with short spinules on both lateral margins. Enp2 covered with short spinules. Laterally with row of spinules on both margins, one outer bipinnate seta and one subterminal inner bipinnate spine. Terminally with a patch of spinules, one short bipinnate seta and one plumose seta.

***P4*** (Fig. 12B). Exopod as in male. Endopod 2-segmented. Enp2 5× longer than Enp1. Enp1 with spinule. Enp2 covered with short spinules. Laterally with row of spinules on both margins below the half-point, one outer bipinnate seta and one subterminal inner bipinnate spine. Terminally with patches of spinules, one short bipinnate seta and one plumose seta.

**Figure 13.**
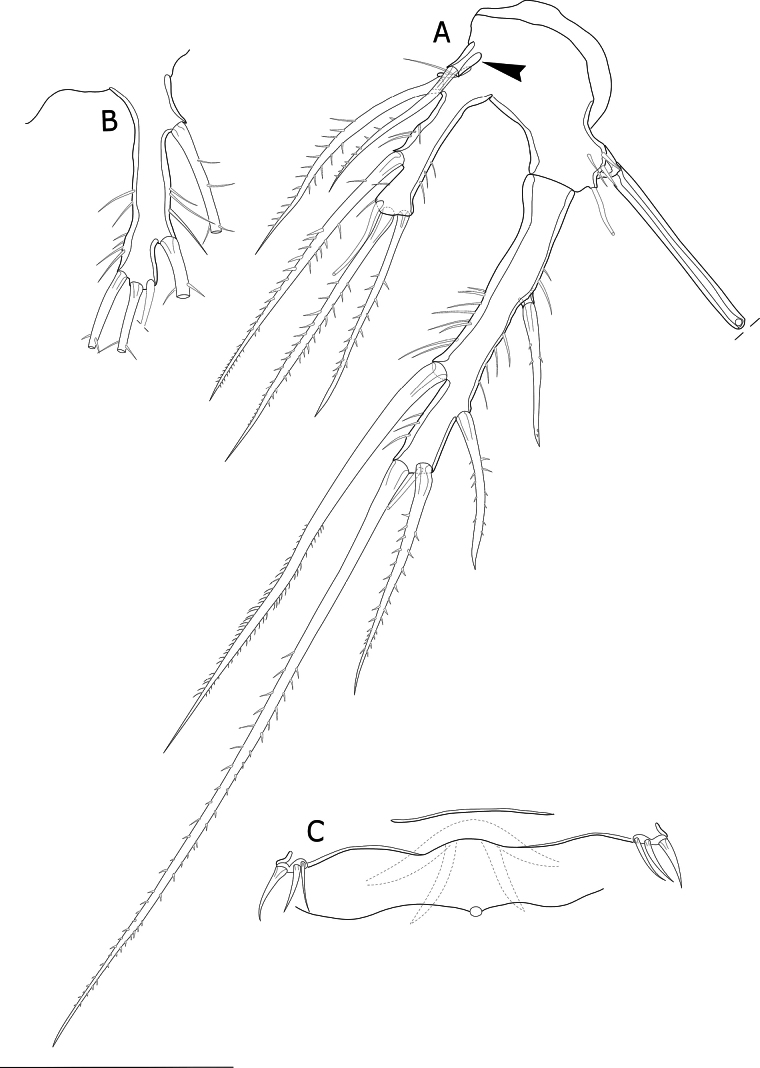
*Breviconia acuminata* sp. nov. ♀ paratype 1 (“allotype”). **A**. Left P5, anterior view. Arrow indicates aberrant pinnate seta on an outgrowth at the baseoendopod; **B**. P5 right endopodal lobe, anterior view; **C**. P6 and genital field. Scale bar: 50 μm.

***P5*** (Fig. 13A, B). Baseoendopod elongated with one outer seta arising from a long setophore which reaches two thirds the length of the exopod. Patch of spinules and one tube pore at the base of the setophore. Endopodal lobe with patches of spinules at both margins and two inner bipinnate spines. Terminally with two bipinnate setae and one tube pore at the inner margin. Exopod 8× as long as wide, with rows of spinules at both margins, one large bipinnate inner seta and two short bipinnate outer spines. Terminally with one long bipinnate seta, one short bipinnate seta and a tube pore in between the two setae.

***P6 and GDS*** (Fig. 13C). P6 small with three bare minute setae, forming the genital operculum. Gonopore located ventrally in the middle of the GDS.

##### Malformations present in examined specimens.

Base of left P5 of paratype 1 (“allotype”) with a short outgrowth near the baseoendopod bearing one terminal pinnate seta (Fig. 13A, arrowed).

**Figure 14.**
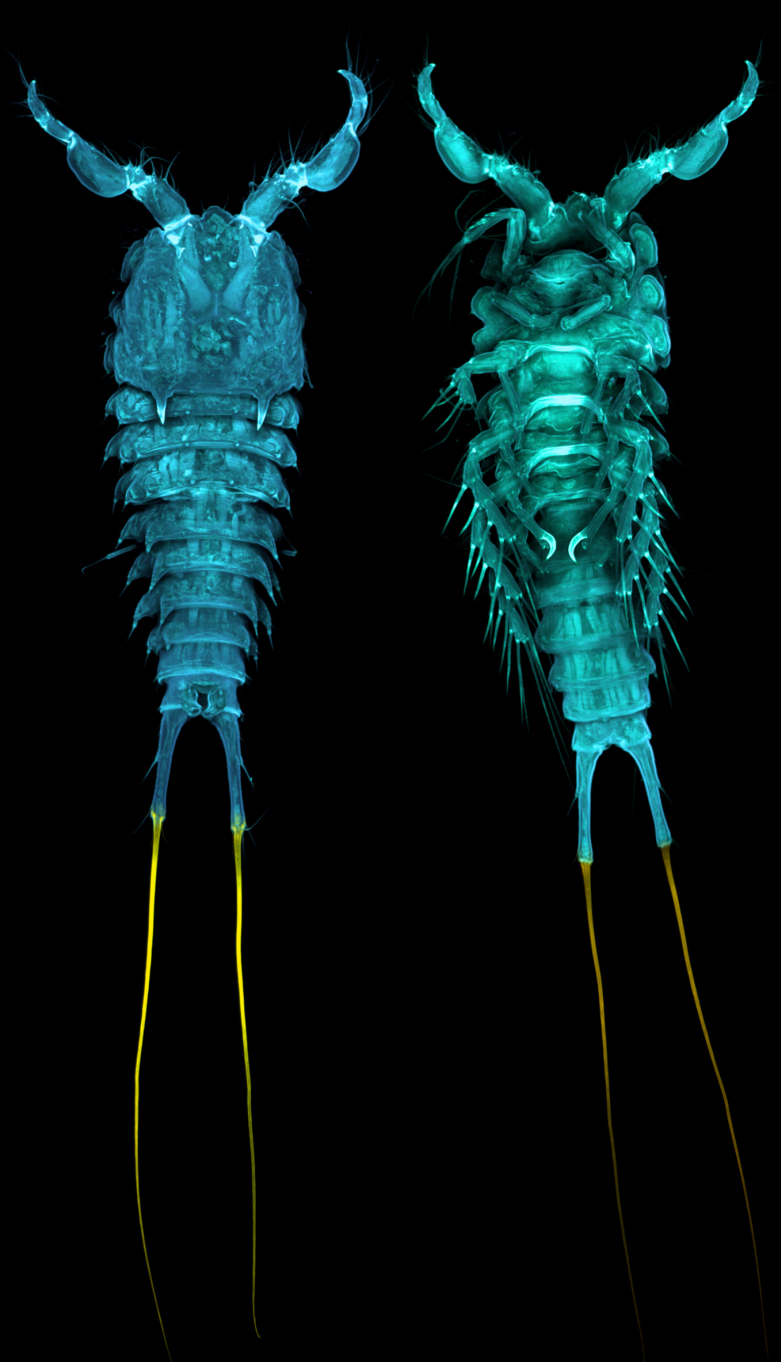
*Breviconia acuminata* sp. nov. ♂ paratype 9. CLSM image. Dorsal, ventral view (left, right). Body length of depicted specimen 541 μm (from anterior tip of rostrum to posterior end of the furcal rami).

**Figure 15.**
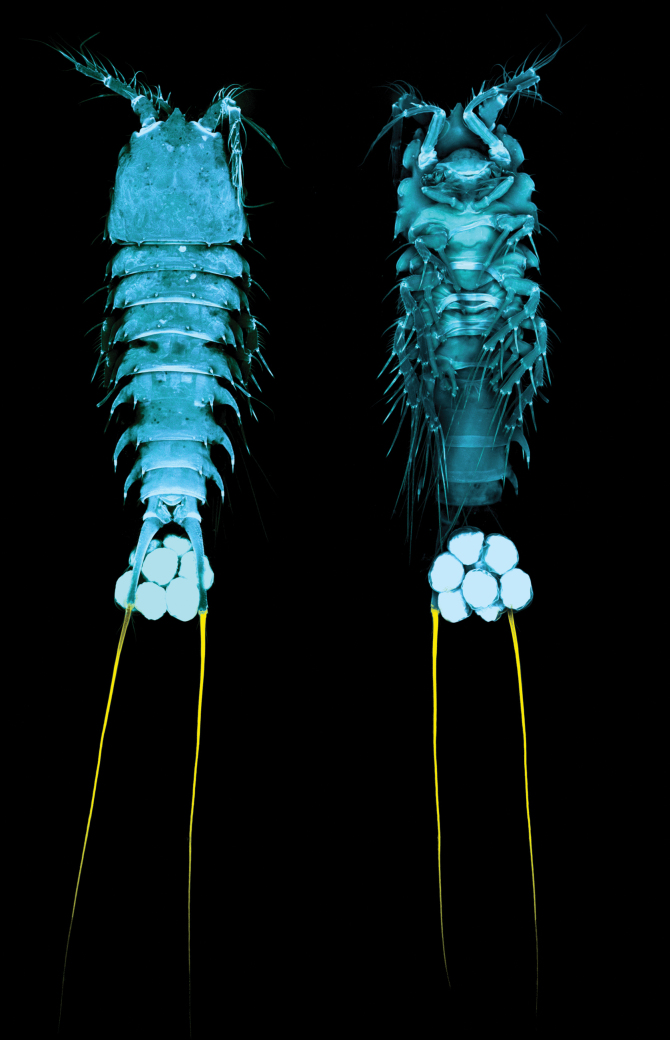
*Breviconia acuminata* sp. nov. ♀ paratype 10. CLSM image. Dorsal, ventral view (left, right). Body length of depicted specimen 665 μm (from anterior tip of rostrum to posterior end of the furcal rami).

Both specimens from station 202 (Bransfield Strait) exhibited morphological variations, but to different degrees. The lateral, dorsolateral, and dorsal cuticular processes, with the exception of the two eponymous processes on the cephalothorax, of male paratype 9 were less well developed than in the other specimens (Fig. 14). All cuticular processes on the cephalothorax and the P2–P5-bearing somites of the female paratype 10 were reduced, with the two eponymous processes on the cephalothorax barely reaching beyond its posterior margin (Fig. 15).

##### Etymology.

The epithet *acuminata* (Latin, meaning sharp, pointed or tapering) refers to the two prominent cuticular outgrowths on the posterior margin of the cephalothorax, which taper towards a tip lacking a terminal sensillum, and to the fact that all specimens were found at stations located near the tip of the Antarctic Peninsula.

##### Phylogenetic analysis.

The assignment of *Breviconia acuminata* sp. nov. to the Ancorabolinae*sensu*George (2020) was clearly possible because the species exhibits all autapomorphic characters of the subfamily (cf. George 2020). Inside the Ancorabolinae, the phylogenetic comparison of the new species was mainly focused on the genera *Arthropsyllus*, *Breviconia* and *Uptionyx* because *B. acuminata* sp. nov. does not share the six autapomorphies of the monophylum *Juxtaramia—Ancorabolus* postulated by Gómez and Conroy-Dalton (2002).

Of the four autapomorphies of a monophylum *Breviconia* assumed by Garlitska et al. (2022), *B. acuminata* sp. nov. shares two [plesiomorphic state in square brackets]:

1. The mx has completely regressed the endopod; it is now represented only by two setae [endopod still present as a small, knob-like segment bearing two setae];
2. The distal endite of the mx now only bears two setae [endite still bears three setae].

Their assignment to *Breviconia* and their monophyletic status can thus be satisfactorily justified, even though two of the criteria listed by Garlitska et al. (2022) no longer apply (see discussion). Within the genus, however, the determination of phylogenetic relationships was somewhat uncertain. First, it was found that each of the three species can be clearly distinguished from the other two by its own derived characters.

*B. andrei*:

1. Female A1 second segment with only seven setae [with at least 8 setae];
2. Female A1 third segment with only 10 setae [with 11 setae];
3. P1Enp1 twice as long as the entire exopod [Enp1 approximately as long as exopod].

*B. australis*:

1. Both inner pairs of sensilla at the posterior margin of the cphth on well-developed conical processes [sensilla arise directly from the smooth cuticle];
2. Basal setophore of P5 significantly longer than the exopod [setophore at most as long as the exopod];
3. Inner seta of P1 basis longer than its Enp1 [inner seta shorter than Enp1].

*B. acuminata*:

1. A1 female: last segment only 9 setae [10 setae];
2. P2Enp2: inner apical seta greatly shortened, only half as long as outer apical seta [both apical setae approximately equal in length].

Within the genus, there is some evidence for a sister-group relationship between *B. australis* and *B. acuminata* sp. nov. Although *B. australis* and *B. andrei* coincide in two exclusive derived characters:

1. Mx distal endite with two setae [with three setae];
2. Mxp claw without small accompanying seta [accompanying seta present],

A clade *B. australis—B. acuminata* sp. nov. can be more clearly established by the following characters:

1. Processes on P2-bearing segment significantly enlarged [processes small];
2. Processes on P3-bearing segment significantly enlarged [processes small];
3. Processes on P4-bearing segment significantly enlarged [processes small];
4. Sensilla on the anal operculum arising from long extensions [extensions short];
5. A1 segments of the female densely covered with small spinules;
6. Mxl coxa with one seta [with two setae].

The sister-group relationship between the two species, which is well supported morphologically by synapomorphies 13–18, is reinforced by their geographical proximity. The clade *B. australis—B. acuminata* sp. nov. is, for its part, the sister group to *B. andrei*, which was described from the Arctic Bering Sea.

### Diagnostic identification key to the *Breviconia* species (females only)

**Table:** 

1	FR cylindrical, slender, < 5× longer than broad; P1Enp1 at most as long as exopod; antennular segments densely covered with minute spinules; A1 2^nd^ segment with 8, 3^rd^ segment with 11 setae	**2**
–	FR trapezoid, tapering distally, ~ 3× longer than broad; P1Enp1 ~ 2× as long as whole exopod; antennular segments lacking dense coverage with minute spinules; A1 2^nd^ segment with 7, 3^rd^ segment with 10 setae	***Breviconia andrei***
2	Sensilla on posterior margin of cphth arising from strong conical processes; female P5 basal setophore longer than exopod; P6 with two setae	***Breviconia australis***
–	Sensilla on posterior margin of cphth originating directly from cuticle; female P5 basal setophore half as long as exopod; P6 with three setae	***Breviconia acuminata* sp. nov**.

## Environment and biogeography

### Geographical, water-column, and sediment characteristics

*Breviconia acuminata* sp. nov. was found at three stations located in the vicinity of the Antarctic Peninsula: Stations 38 and 120 in the north-western Weddell Sea and station 202 west of this region in Bransfield Strait. The distance between stations 38 and 120, which are in close proximity, and station 202 was ca 190 km (Fig. 1). While the two stations in the Weddell Sea were clearly situated on the Antarctic shelf (at water depths of 427 and 504 metres, respectively), station 202, with a water depth of 757 metres, was located in a trough that opens onto the continental slope (Table 1; Veit-Köhler et al. 2018).

Salinity near the seafloor was similar at all three stations and fell within the range observed during expeditions PS 81, PS 96, and PS 118 (34.45–34.67). However, water temperature near the seafloor, *_bottom_*T, clearly differed between the geographically close stations 38 and 120. This difference was probably due to the fact that sampling at station 38 took place end of March (–0.86 °C) while sampling at station 120 (–1.81 °C) was conducted end of January, in early summer (Table 3; Schröder et al. 2013; Janout et al. 2020).

**Table 3.** Environmental parameters measured at stations where *Breviconia acuminata* sp. nov. was found. North-western Weddell Sea: st. 38 (RV Polarstern expedition PS 118), st. 120 (PS 81); Bransfield Strait: st. 202 (PS 81). Sediment parameters averaged from 0–5 cm sediment depth. For PS 118, pigment measurements were obtained from the same two cores in which *B. acuminata* sp. nov. was detected. All other data retrieved from cores from the same MUC deployments. Abbreviations used: Water-column parameters – *_bottom_*: measurement close to the seafloor; T: water temperature; Sal: salinity. Sediment parameters – *_sed_*: parameter measured from sediment samples; Chl *a*: content of chlorophyll *a*; Phaeo: content of phaeopigments; C/N*_molar_*: molar carbon:nitrogen ratio; silt&clay: grain size fractions <63 μm; sand: 63–500 µm; coarse sand: >500 µm.

Environmental parameter	PS 81 st. 120-5	PS 81 st. 202-5	PS 118 st. 38-3
*_bottom_*T [°C]	–1.81	–0.72	–0.86
*_bottom_*Sal	34.49	34.55	34.57
*_sed_*Chl *a* [µg g^-1^]	29.31	2.13	4.00
*_sed_*Phaeo [µg g^-1^]	14.66	6.76	11.67
*_sed_*C/N*_molar_*	6.70	5.42	9.41
silt&clay %	88.92	86.11	84.29
sand %	10.82	13.59	15.71
coarse sand %	0.26	0.29	0

The grain size composition was similar at all three stations with a clear dominance of the silt and clay fraction (84.3–88.9%). The chloroplastic equivalents (CPE = sum of the content of chlorophyll *a _sed_*Chl *a* and phaeopigments *_sed_*Phaeo) were high at station 38 and very high at station 120 (15.67 and 43.97 µg g^-1^, respectively) but much lower at station 202 (8.89 µg g^-1^). On the other hand, the molar Carbon:Nitrogen ratio, *_sed_*C/N*_molar_*, an indicator for the freshness of the organic material in the sediment, was low at stations 120 and 202 (6.7 and 5.4, respectively) and high at station 38 (9.4). A higher value indicates more degraded material at this station (Table 3; Vanreusel et al. 2021; Weith et al. 2023).

### Abundance of meiofauna and *Breviconia acuminata* sp. nov.

In the course of the three expeditions PS 81, PS 96, and PS 118 altogether 102 sediment cores were collected for meiofauna analyses (PS 81: 47 cores; PS 96: 42 cores; PS 118: 13 cores; Veit-Köhler et al. 2017; Säring et al. 2021; Corus et al. 2024; unpubl. data). For the vast majority of the samples, copepods (adults and copepodids) were picked and transferred to depression slides with glycerine so that they could be examined for the presence of *Breviconia acuminata* sp. nov. In total, 41,220 copepod specimens were visually inspected (PS 81: 20,277 ind.; PS 96: 8,623 ind.; PS 118: 12,320 ind.). Only 11 adult specimens of the new species were found. In the four cores that contained *Breviconia acuminata* sp. nov., the species was encountered in 0–1 or 1–2 cm sediment depth and reached densities of 0.2–0.9 ind. 10 cm^-2^ (Table 1).

Individual densities of nematodes in these four cores were in the range of densities usually encountered in the north-western Weddell Sea (1,691.0–7,957.3 ind. 10 cm^-2^) and Bransfield Strait (1,443.5–5,700.8 ind. 10 cm^-2^; Veit-Köhler et al. 2017; Säring et al. 2021; Corus et al. 2024). The number of copepods per 10 cm^2^ was exceptionally high at stations 38 and 120 (max. values of 940.4 and 916.5 ind. 10 cm^-2^, respectively). Only one more station, as well situated in the northernmost investigated area, held nearly as high copepod densities (741.2 ind. 10 cm^-2^, st. 163, PS 81). Station 202 in Bransfield Strait, which contributed two specimens of *Breviconia acuminata* sp. nov., had a medium to low copepod density (89–140 ind. 10 cm^-2^) which was in line with other stations in Drake Passage and Bransfield Strait. Over all other sediment cores investigated from expeditions PS 81, PS 96, and PS 118 (Veit-Köhler et al. 2017; Säring et al. 2021; Corus et al. 2024; unpubl. data) the maximum copepod density was 277.4 ind. 10 cm^-2^ (PS 118, st. 8).

## Discussion

### Phylogenetic analysis

*Breviconia acuminata* sp. nov. can undoubtedly be assigned to the Ancorabolinae*sensu*George (2020). Based on the phylogenetic analysis by Conroy-Dalton and Huys (2000), George (2020, Table 1) listed 12 autapomorphies of the subfamily; five of which relate to the complex “sensillar groups” I–V on the Cphth recognised by Conroy-Dalton and Huys (2000). Each of these “sensillar groups” comprises a number of sensilla that arise from more or less pronounced outgrowths of the cuticle. In addition, there are seven further characters, such as the formation of lateral cuticular processes on most body segments and the development of special sensilla on these processes (cf. George 2020: Table 1). *B. acuminata* sp. nov. shares all 12 autapomorphies.

It was not the intention of the present study to clarify the phylogenetic relationships within the Ancorabolinae. An initial morphological comparison of all related species carried out in advance has already shown that the relationships are complex and require extensive investigation, which cannot be accomplished in the present work. *Breviconia acuminata* sp. nov. and the other two *Breviconia* species share a whole series of derived characters, many of which, however, occur scattered among representatives of the other genera and are therefore not exclusive autapomorphies of *Breviconia*. Ongoing studies are devoted to the phylogenetic relationships within the Ancorabolinae and will be published elsewhere.

Despite the fact that they are the only two characters remaining of the four postulated by Garlitska et al. (2022), characters 1 and 2 allow *Breviconia* to be identified as a monophyletic group. Apomorphy 1, the completely reduced endopod of the maxilla, represented only by two setae, is found exclusively in *Breviconia*; all other Ancorabolinae still possess the tiny but clearly recognisable endopod of Cletodoidea Bowman & Abele, 1982, which bears the two setae (cf. George 2020: Table 1, character 31). Apomorphy 2, the possession of only two setae on the distal endite of the maxilla, is also an exclusive and derived feature of *Breviconia*. All other representatives of the Ancorabolinae retain three setae (as well as on the proximal endites), and even within the Ancorabolidae themselves, there is only one other taxon, *Probosciphontodes* Fiers, 1988 (Laophontodinae Lang, 1944), in which only two setae occur on the two maxillary endites (Fiers 1988).

The remaining two autapomorphies of the genus suggested by Garlitska et al. (2022) – the reduction of a seta on the proximal maxillary endite and the loss of the tiny accompanying seta on the claw of the maxilliped – must be rejected with the discovery of *B. acuminata* sp. nov., as the species exhibits the plesiomorphic state in both characters: it still has the third seta on the proximal maxillary endite (Fig. 5E) and also the small accompanying seta on the maxilliped (Fig. 5F). The presence of the third seta on the proximal endite shows once again how important it is to identify characters as clearly as possible and to avoid summarising characters. Character 3 of Garlitska et al. (2022) – the endites of the maxilla bear only two setae instead of three – is in fact a complex of two characters, because each endite must be considered separately.

In our opinion, the search for autapomorphies of individual species and higher taxa is a very important aspect of phylogenetic analyses, because only in this way is it possible to characterise a taxon phylogenetically in an unambiguous manner, rather than merely typologically, for which all diagnostic features are helpful. We expressly disagree with Gómez and Conroy-Dalton (2002), who argued that autapomorphies have no phylogenetic value and merely increase the length of a cladogram. For this reason, it was important to us to clearly characterise and distinguish the three representatives of *Breviconia* on the basis of autapomorphies. The listed autapomorphies (*B. andrei*: characters 3–5; *B. australis*: characters 6–8; *B. acuminata* sp. nov.: characters 9 and 10) are essentially self-explanatory: the respective species exhibits the derived state, whereas the other two species retain the plesiomorphic state. Regarding character 5, we assume that the elongation of P1Enp1 in *B. andrei* is indeed a derivation. Basically, we follow George’s (2020: 469 ff.) hypothesis that the ancorabolid P1 developed from the prehensile podogennontan P1 and thus evolved an elongated Enp1 (George 2020, fig. 3). This author further concluded that the non-prehensile P1 of the Cletodidae, which bears a short Enp1, is a further derivation of the original podogennontan P1. Nonetheless, the extent to which this assumption is also reflected within the Ancorabolinae will have to be clarified in the context of a comprehensive phylogenetic analysis of that subfamily. After all, *Arthropsyllus serratus*, which is considered the most primitive representative of the Ancorabolinae, has an elongated P1Enp1, but it is shorter than that of all other ancorabolin species. For the time being, we follow the hypothesis of Conroy-Dalton and Huys (2000) of a “progressive elongation” of the P1Enp1 in the Ancorabolinae; however, this assumption will also have to be tested in the context of the ongoing phylogenetic analysis of the subfamily.

It came as no surprise that *Breviconia australis* and *B. acuminata* sp. nov. are more closely related than *B. australis* and *B. andrei*. Although the sister-group relationship between *B. australis* and *B. andrei* hypothesised by Garlitska et al. (2022) was well justified by four synapomorphies, only these two species were known at the time. Furthermore, of the four synapomorphies, only two derived characters remain (characters 11 and 12), which, however, must be regarded as convergent developments due to the much greater similarity in derived characters between *B. australis* and *B. acuminata* sp. nov. (characters 13–18). Considering that characters 11 and 12 are reductions of setae – a widespread process in Harpacticoida –, the assumption of convergence is more plausible than that of synapomorphic development. This is more likely to be the case for characters 13–18; five of them involve the emergence of new structures (elongation of the dorsal processes on the P2–P4-bearing somites; extension of the sensilla-bearing bases on the anal operculum; dense coverage with small spinules on the segments of the female A1), i.e. much more complex derived characters than the simple reduction of elements. To assume their convergent development, especially in species that occur relatively close to each other geographically (Magellan Region and Antarctic Peninsula), seems much less plausible than the hypothesis that these characters are synapomorphies of sister species. The only reservation in this context is the fact that apomorphy 17 is also found in the females of *Juxtaramia polaris* Conroy-Dalton & Huys, 2000 and *Ancorabolus inermis* Conroy-Dalton & Huys, 2000 and even *Uptionyx verenae* Conroy-Dalton & Huys, 2000 has a patch of small spinules on the first segment of the A1. Thus, character 17 must also be subjected to careful examination in a comprehensive phylogenetic analysis of the Ancorabolinae.

Finally, we must address the supposed elongation of the mandibular gnathobase and its curvature of nearly 90°, cited as character 1 by Garlitska et al. (2022). This character was first described by George (1998: fig. 3E) for *B. australis*, and it was also documented in *B. andrei* (Garlitska et al. 2022: fig. 4C). Garlitska et al. therefore assumed that this was a synapomorphy shared by *B. australis* and *B. andrei*. However, Conroy-Dalton and Huys (2000) had already qualified George’s (1998) assumption by making the representation of a curvature such as that in *B. australis* dependent on the position of the mandible on the slide. And indeed, George (2001: fig. 4A, B) had documented a similarly strongly curved gnathobase of the mandible in *Ancorabolus ilvae* George, 2001. However, a second drawing he made depicted the mandible as described for most representatives of the Ancorabolinae and also for *B. acuminata*, where the curvature is not noticeable. We must therefore conclude that Conroy-Dalton and Huys (2000) are correct, and that means that character 1 of Garlitska et al. (2022) must therefore be rejected.

### Role of the environment

In the Antarctic summer, decaying phytoplankton and ice algae released by melting sea ice sink to the seafloor. Due to the low temperatures, this organic material is not fully consumed and forms food banks, which are an important feature of the Antarctic benthos in the seasonal sea-ice zone (Mincks et al. 2005). High amounts of phytodetritus (CPE) at stations 120 and 38 are the result. While the high sediment C/N molar ratio at station 38 (9.4) suggests the presence of more degraded material (probably due to the late sampling date in March), the low ratio at stations 120 and 202 indicates the presence of fresh organic material in the sediment (Veit-Köhler et al. 2018; Säring et al. 2022b; Corus et al. 2024). Thus, the benthic communities at stations 120 and 38 are supplied with food year-round, which suggests that *Breviconia acuminata* sp. nov. prefers areas with abundant food sources.

### Distribution ranges and sampling effort

Despite extensive sampling, the study of material from three expeditions, and microscopic examination of tens of thousands of benthic copepods, *Breviconia acuminata* sp. nov. was found only in small numbers and in a very limited area at the northernmost tip of the Antarctic Peninsula and in Bransfield Strait. Its congener, *Breviconia australis*, was described from a single station in the eastern Beagle Channel (Chile, 55°00.4'S, 66°53.6'W, st. 1234/I, ‘Magellan Campaign’ of RV VICTOR HENSEN, 18.11.1994) at a depth of 100 m (George 1998). Despite investigating 20 stations in total in the Straits of Magellan, the Beagle Channel, and on the Patagonian continental slope, George (2005) found only one specimen of *Breviconia australis* (see his table 4).

Very limited distribution areas are not uncommon among representatives of marine benthic Harpacticoida. Other examples of species from the region with only local references despite extensive sampling are *Laophontodes gertraudae* George, 2018 and *Laophontodes sabinae* George & Gheerardyn, 2015 (Ancorabolidae) as well as *Gideonia noncavernicola* George & Martínez Arbizu, 2005 (Superornatiremidae) and *Isthmiocaris longitelson* George & Schminke, 2003 (Canthocamptidae). They all originate from the Patagonian continental slope south of Isla Nueva (George and Schminke 2003; George and Martínez Arbizu 2005; George and Gheerardyn 2015; George 2018). In the Weddell Sea, *Emertonia berndi* Mathiske & Veit-Köhler, 2021 (Paramesochridae) was found in high numbers (12 individuals) but only at two stations from the same location. However, the same large-scale study, which spanned three oceans, also described three other species of the same genus that had much larger distribution ranges (Mathiske et al. 2021).

Sometimes, only few specimens of a new species are found during an entire expedition. One example is *Dendropsyllus antarcticus* (George & Schminke, 1998) (Ancorabolidae), a species based on a single female specimen collected in Halley Bay, Antarctica (George and Schminke 1998). However, species that were initially described based on a small number of individuals from a limited geographical area may be found in other locations when the search is expanded, albeit always in small numbers. The first and only female specimen of *Dendropsyllus magellanicus* (George & Schminke, 1998) was collected in Paso Ancho in the Strait of Magellan (George and Schminke 1998). A corresponding male specimen was found and described years later off Chiloé, almost 1,200 km away (George 2006). Another example is *Emertonia andeep* (Veit-Köhler, 2004), which was described based on two females collected in the Weddell Sea (Veit-Köhler 2004). Subsequently, it was discovered that, although this species is always rare, it is widely distributed in the south-eastern Atlantic (Gheerardyn and Veit-Köhler 2009: fig. 2, previous species name *Kliopsyllus andeep*).

The local distribution range of *Breviconia acuminata* sp. nov. raises questions that cannot be fully answered within the scope of this study. (1) Was the very rare new species detected at the tip of the Antarctic Peninsula only because of the extraordinarily high copepod densities present in this area? Station 163 (WS-ET-D in Veit-Köhler et al. 2018) in the north-western Weddell Sea had copepod densities that were almost as high (741.2 ind. 10 cm^-2^). However, the copepods from this station could not be examined as the meiofauna was counted and deposited at the University of Ghent (Hauquier et al. 2015). The other location that held *B. acuminata* sp. nov. (station 202 in Bransfield Strait) showed medium to low copepod densities. (2) Could undersampling in low-density areas explain why the new species was not found at more stations? Manually sorting meiofauna samples and picking individuals for morphological inspection is time-consuming. It took years of work by many students and technicians to obtain the material for further analysis. Therefore, splitting samples or investigating only selected cores is sometimes the only option when faced with time constraints. Finally, (3) the distribution range of the new species may be restricted to zones with high seasonal changes in sea-ice cover, as it may prefer exceptionally high food availability, as observed at stations 38 and 120. However, most of our samples were taken from areas where the conditions were not as favourable (Veit-Köhler et al. 2018; Säring et al. 2022b; Weith et al. 2023).

Further studies will involve metabarcoding of whole meiofauna communities and barcoding of single copepod specimens. These studies may clarify the true distribution range of *Breviconia acuminata* sp. nov., which may require revision in the future.

## Conclusions

*Breviconia acuminata* sp. nov. is a rare benthic copepod that has a limited distribution range on the Antarctic continental shelf. The majority of the specimens were found at two stations at the tip of the Antarctic Peninsula, which were characterised by high food availability and extremely high copepod densities.

With the discovery of *Breviconia acuminata* sp. nov., a third species could be added to the genus *Breviconia*. The significance of this discovery can be gauged by the fact that *Breviconia acuminata* sp. nov. provided new morphological characters that allowed for a more precise phylogenetic characterisation of the monophylum *Breviconia*. Even if not all four autapomorphies proposed by Garlitska et al. (2022) remain valid, the currently known three species can still be grouped together in *Breviconia* on the basis of two synapomorphies. This is particularly noteworthy because *Breviconia* appears to have a very wide and at least bipolar distribution range, whereas the respective species have so far shown only localised distribution. However, our morphological comparison clearly demonstrates a closer relationship between the two (sub)Antarctic species *B. australis* and *B. acuminata* sp. nov., which together form the sister group of the Arctic counterpart *B. andrei*. Future studies should bring clarity to these disjunctive distribution patterns.

## Supplementary Material

XML Treatment for Breviconia

XML Treatment for Breviconia acuminata

## Additional information

### Artificial Intelligence (AI) use

The authors accept full responsibility for the content of the manuscript, including the disclosure of any use of AI.

Regarding the use of AI in the preparation of this manuscript, the authors declare the following: DeepL.

Used for: Other.

DeepL Write was used to check the English grammar and style.

### Author ORCIDs

J.C. Nierste https://orcid.org/0009-0000-7484-0289

G. Veit-Köhler https://orcid.org/0000-0002-3453-898X

K.H. George https://orcid.org/0000-0001-6464-0099

### Data availability

All data supporting the findings of this study are available in the main text and the cited data sets.
